# 3D-organization and spatial localization of chromatin and epigenetic marks linked to nucleolar activity in porcine oocytes^†^

**DOI:** 10.1093/biolre/ioaf098

**Published:** 2025-05-03

**Authors:** Merle Fenner, Michal Benc, Alexandra Rosenbaum Bartkova, Maria Pihl, Martine Chebrout, Martine Letheule, Frantisek Strejcek, Poul Hyttel, Jozef Laurincik, Kristine Freude, Amelié Bonnet-Garnier

**Affiliations:** Faculty of Health and Medical Sciences, Department for Veterinary and Animal Sciences, Section Pathobiological Sciences, University of Copenhagen, Copenhagen, Denmark; Faculty of Natural Sciences and Informatics, Constantine the Philosopher University in Nitra, Nitra, Slovakia; Faculty of Natural Sciences and Informatics, Constantine the Philosopher University in Nitra, Nitra, Slovakia; Institute of Animal Physiology and Genetics, The Czech Academy of Sciences, Libechov, Czech Republic; Faculty of Health and Medical Sciences, Department for Veterinary and Animal Sciences, Section Pathobiological Sciences, University of Copenhagen, Copenhagen, Denmark; Université Paris-Saclay, UVSQ, INRAE, BREED, 78350, Jouy-en-Josas, France; Ecole Nationale Vétérinaire d’Alfort, BREED, 94700, Maisons-Alfort, France; Université Paris-Saclay, UVSQ, INRAE, BREED, 78350, Jouy-en-Josas, France; Ecole Nationale Vétérinaire d’Alfort, BREED, 94700, Maisons-Alfort, France; Faculty of Natural Sciences and Informatics, Constantine the Philosopher University in Nitra, Nitra, Slovakia; Faculty of Health and Medical Sciences, Department for Veterinary and Animal Sciences, Section Pathobiological Sciences, University of Copenhagen, Copenhagen, Denmark; Faculty of Natural Sciences and Informatics, Constantine the Philosopher University in Nitra, Nitra, Slovakia; Faculty of Health and Medical Sciences, Department for Veterinary and Animal Sciences, Section Pathobiological Sciences, University of Copenhagen, Copenhagen, Denmark; Université Paris-Saclay, UVSQ, INRAE, BREED, 78350, Jouy-en-Josas, France; Ecole Nationale Vétérinaire d’Alfort, BREED, 94700, Maisons-Alfort, France

**Keywords:** oocyte developmental biology, epigenetics, porcine oocytes, chromatin conformation, pericentromeric satellite DNA, transcriptional activity, nucleolar sphere, brilliant cresyl blue

## Abstract

Previous murine studies have established that large-scale chromatin modifications upon completion of oocyte growth are associated with nucleolar transcriptional silencing. These modifications seem essential both for completion of the oocyte’s meiosis and subsequent embryonic developmental success. Investigating this putative interconnection between nucleolar transcriptional activity and spatial chromatin organization towards completion of oocyte growth in pigs, we scrutinized whether 3D chromatin organization and heterochromatin localization, along with epigenetic markers, could indicate oocyte quality and predict developmental competence of harvested porcine oocytes. Supravital brilliant-cresyl-blue staining was used to classify porcine cumulus-oocyte-complexes as fully grown (brilliant-cresyl-blue+) or still growing (brilliant-cresyl-blue−). Oocytes were analyzed via integrated 3D-immunofluorescence for nucleolar activity and heterochromatin markers, as well as 3D-DNA-fluorescence in situ hybridization for specific heterochromatin sequences. Additionally, some oocytes were prepared for transmission electron microscopy. Transmission electron microscopy revealed distinct ultrastructural differences between brilliant-cresyl-blue+ and brilliant-cresyl-blue− oocytes and validated brilliant-cresyl-blue-staining as viable method for a rough assessment of oocyte developmental competence. Immunostaining identified all known germinal vesicle chromatin configurations (non-surrounded nucleolus, partially non-surrounded nucleolus, partially surrounded nucleolus, surrounded nucleolus) and linked them to respective brilliant-cresyl-blue categories. Nucleolar activity was detected only in non-surrounded nucleolus oocytes, predominantly from the brilliant-cresyl-blue− group. Protein markers and fluorescence in situ hybridization signals revealed significant 3D-organizational changes in chromatin between non-surrounded nucleolus and surrounded nucleolus conformations, clustering around the nucleolus towards final oocyte maturation. These findings highlight an evident interconnection between nucleolar transcriptional silencing and specific 3D chromatin organization patterns, with changes in heterochromatin localization indicating completion of the oocytes’ growth phase and marking higher competency for subsequent final maturation and embryonic development.

## Teaser

**Summary Sentence** Utilizing 3D-immunolocalization and high-resolution microscopy, chromatin-specific epigenetic marks and proteins specific to nucleolar activity can be linked to oocyte maturation, predicting subsequent embryonic developmental competence.

## Graphical Abstract

**Graphical Abstract:**
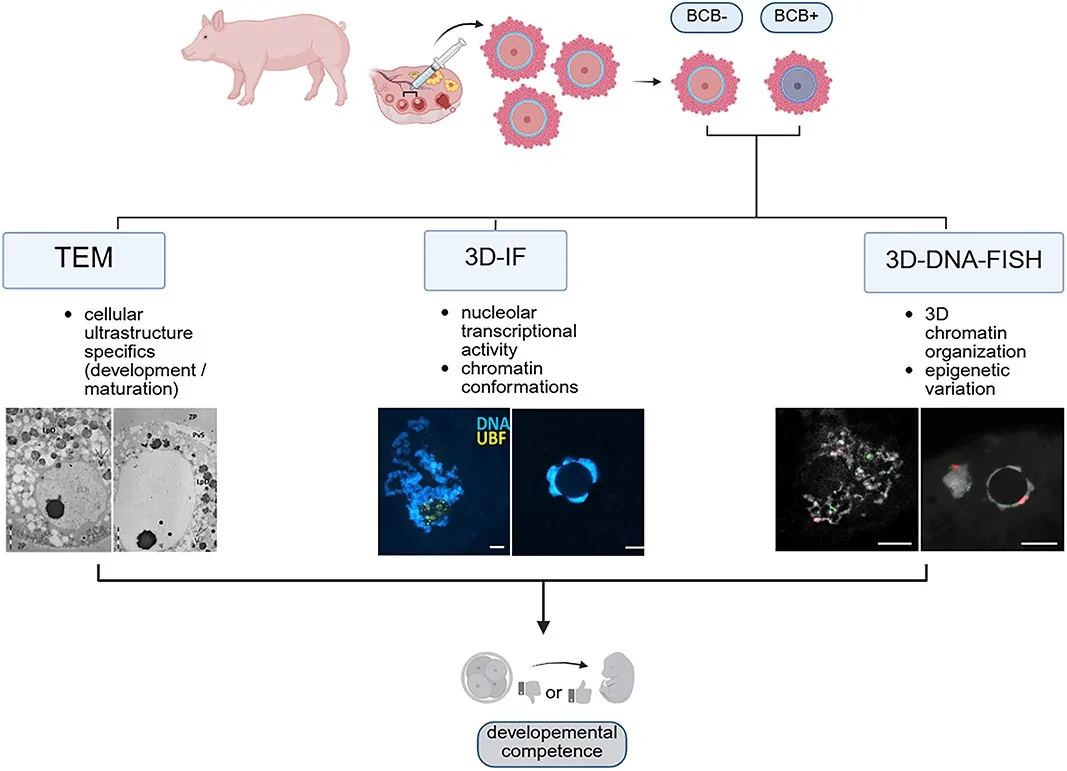

## Introduction

As the use of assisted reproductive technologies (ARTs) increases worldwide [1], enhancing the quality of oocyte selection for embryo technologies such as in vitro fertilization and embryo production is essential to maximize success. This imperative applies not only to human but also veterinary reproductive medicine [2–5]. Poor oocyte quality has been associated with reduced success rates in in vitro maturation, fertilization, and embryo development [6, 7], underscoring the critical role of oocyte quality in advancing the effectiveness of ARTs and infertility treatments.

Given the close resemblance between porcine oocyte morphology, biology, and embryonic development in their human counterpart, the potential of this species for translational research in oocyte quality and subsequent embryonic development is evident [8].

However, the existing literature reveals a distinct knowledge gap regarding the growth and maturation processes and the organization of chromatin and epigenetic marks in porcine oocytes compared to commonly acknowledged model species such as mice.

Furthermore, non-invasive, supravital selection methods proposed to date, do not fully comprehend the multifactorial complexity of oocyte growth, maturity, and quality [9], reflected by the continued reliance on morphological characteristics in oocyte quality selection processes [10].

Employing immunolocalization and high-resolution microscopy analyses of chromatin configurations, chromatin-specific epigenetic marks, and proteins specific for nucleolar activity, the present study aims to address the apparent knowledge gap by enlarging upon chromatin remodelling linked with the transcriptional arrest underlying the completion of porcine oocyte growth. Furthermore, we seek to investigate how an existing method of supravital oocyte selection e.g. brilliant cresyl blue (BCB) staining correlates with three-dimensional (3D) chromatin organization in porcine oocytes.

BCB-staining is one of the most used and investigated techniques for non-invasive supravital selection of mammalian oocytes in ARTs [2, 3, 11–15]. However, its use has been controversial for decades [12, 16, 17]. In short, this staining is based on the unique characteristics of oocyte glucose metabolism, including the pentose phosphate pathway and its rate-controlling enzyme glucose-6-phophate dehydrogenase (G6PDH). G6DPH activity has become a well-recognized marker of cytoplasmic maturation in oocytes [18]. The enzyme is able to metabolize the blue BCB dye and as its activity decreases towards the end of oocyte growth and maturation, it is suitable for selection between fully grown, and hence competent, BCB positive (BCB+) and still growing, and hence less competent, BCB negative (BCB−) oocytes [13, 14], and therefore an appropriate quality pre-selector for the biological material in this study.

Previous studies in several mammalian species have confirmed that the nucleolus of the oocyte is subjected to large-scale modifications correlated to transcriptional silencing during final maturation. These modifications seem essential both for completion of the oocyte’s meiosis and subsequent embryonic developmental success [19–24]. In mice, these nucleolar events have been linked to sequential changes in spatial 3D-organization of the chromatin towards completion of the oocyte growth phase [23]. In the porcine species, however, knowledge about the spatial heterochromatin organization and nucleolar transcriptional activity towards completion of oocyte growth are still lacking.

To allow for direct relation of both porcine and murine data, we employed the classification for porcine germinal vesicles (GV), published by Pan et al [24], describing the sequential changes in spatial chromatin conformation in relation to the nucleolus of the oocyte: from non-surrounded nucleolus (NSN) to surrounded nucleolus (SN) and their intermediate forms partially non-surrounded nucleolus (pNSN) and partially surrounded nucleolus (pSN).

Bonnet-Garnier et al. [23] have examined the distribution of epigenetic marks related to euchromatin (histone post-translational modifications linked to active transcription like H3K4me3, [23]) and heterochromatin, like H3K9me3 and centrometric proteins (CENPs) during the NSN to SN transition in mice. They established that centromeres and their surrounding pericentromeric regions form so called chromocenters, which are dispersed throughout the nucleoplasm, in NSN type oocytes. SN oocytes, however, display a significant decrease in chromocenters and a relocation of heterochromatin around the nucleolar sphere (NS).

Based on these findings, we chose to investigate the spatial localization within the nucleoplasm of histone 3 (H3) lysine 9 three methylation (H3K9me3) and of histone variants of centromeric protein A and B (CENPs), as well as porcine meta- and acrocentric heterochromatin sequences using specific oligonucleotide probes, and thereby overall 3D chromatin organization throughout the later stages of the porcine oocytes’ growth phase [25]. We additionally included upstream binding factor (UBF) as a marker for nucleolar transcriptional activity [26], as its expression is vital for the functionality of the nucleolar transcription initiation complex (TIC; moreover, consisting of RNA polymerase I and promoter selectivity factor 1) and thereby transcription of ribosomal DNA (rDNA) within the nucleolus. Furthermore, we chose to support the visualization of potential changes in transcriptional status and euchromatin organization of growing porcine oocytes by modified fluorescent staining of newly transcribed RNA via native incorporation of 5-ethynyluridine (EU) [27] in combination with histone 4 (H4) lysine 16 acetylation (H4K16ac) and UBF, as well as histone 3 (H3) lysine 4 trimethylation (H3K3me3), marking transcriptional activation [28].

Utilizing fluorescence-in situ- hybridization (FISH) and integrated immunofluorescence (IF) techniques on three-dimensionally (3D) preserved oocytes analyzed by high-resolution microscopy, the present study was designed to investigate the putative interconnection between nucleolar transcriptional activity and spatial chromatin organization towards completion of oocyte growth. By linking the progression and completion of oocyte growth to embryonic developmental competence in porcine oocytes, we expect to determine whether the 3D-organization and spatial localization of chromatin and epigenetic marks can function as diagnostically conclusive indicators to predict the final developmental competence of harvested oocytes in relation to their initial developmental status.

## Material and methods

All chemicals were purchased from Sigma-Aldrich, unless stated otherwise.

### Ethical statement

The handling of biological material used in this study was conducted in accordance with Good Laboratory and Scientific Practice (GLP and GSP) statements of all involved research institutions. As the studied porcine oocytes were obtained from a local abattoir, no further review of local Animal Experiment Inspectorates was required.

### Recovery of biological material

Porcine ovaries were collected from post pubertal gilts (8–10 months of age) at a local abattoir. The freshly collected ovaries were stored in a thermos container at 38°C and transported in pre-warmed sterile phosphate-buffered saline (PBS) within 1 h to the laboratory.

#### Oocyte selection

As recently described by Bartkova et al [5], cumulus-oocyte-complexes (COCs) were aspirated from 3 to 6 mm follicles, classified as healthy in accordance with Liu et al. [29], using a 21G x 1 ½ ″ needle and 10 ml syringe. The collected aspirate was allowed to settle for 20 min at room temperature. The sediment was then used for COC collection under a Nikon SMZ800N stereomicroscope (Tokyo, Japan, 45× magnification). COCs were recovered, collected, and washed three times in modified PBS (mPBS) medium (1xPBS supplemented with 0.4% bovine serum albumine (BSA), 0.34 mM glucose, and 5.5 mM sodium pyruvate [22]). Complexes deemed adequate to include into the study, were selected after morphological criteria like compactness of the cumulus investment and ooplasm characteristics [30].

#### Supravital BCB staining, oocyte classification, and fixation

The selected COCs were incubated in mPBS supplemented with 13 μM BCB for 90 min. Following incubation, the COCs were washed in mPBS, observed under a Zeiss LSM 710 microscope (Zeiss, Germany) with phase contrast, and divided into two qualitative subgroups, classified as BCB positive (+) or BCB negative (−), referring to their respective BCB staining (Figure 1B). As previously stated in the introduction, this classification method is relying on BCB dye, as supravital indicator for cytoplasmatic G6PDH activity and its relation to cytoplasmic maturation status and developmental capacity of the oocyte [11, 14]. Subsequently, the oocytes were incubated in 1× PBS supplemented with 0.3% hyaluronidase from bovine testes, and mechanically denuded from the surrounding cumulus cells by gentle pipetting and placed in pre-warmed (37°C) M2 medium for a few seconds. Whereafter they were transferred to 300 μl of Tyrode for 60 s at room temperature (RT) and in the following washed in 500 μl M2 medium supplemented with 5 μl phenylmethylsulfonyl fluoride (PMSF93482, Fluka). The last washing step prior to fixation was conducted with 0.5% PVP/PBS supplemented with 5 μl PMSF.

**Figure 1:**
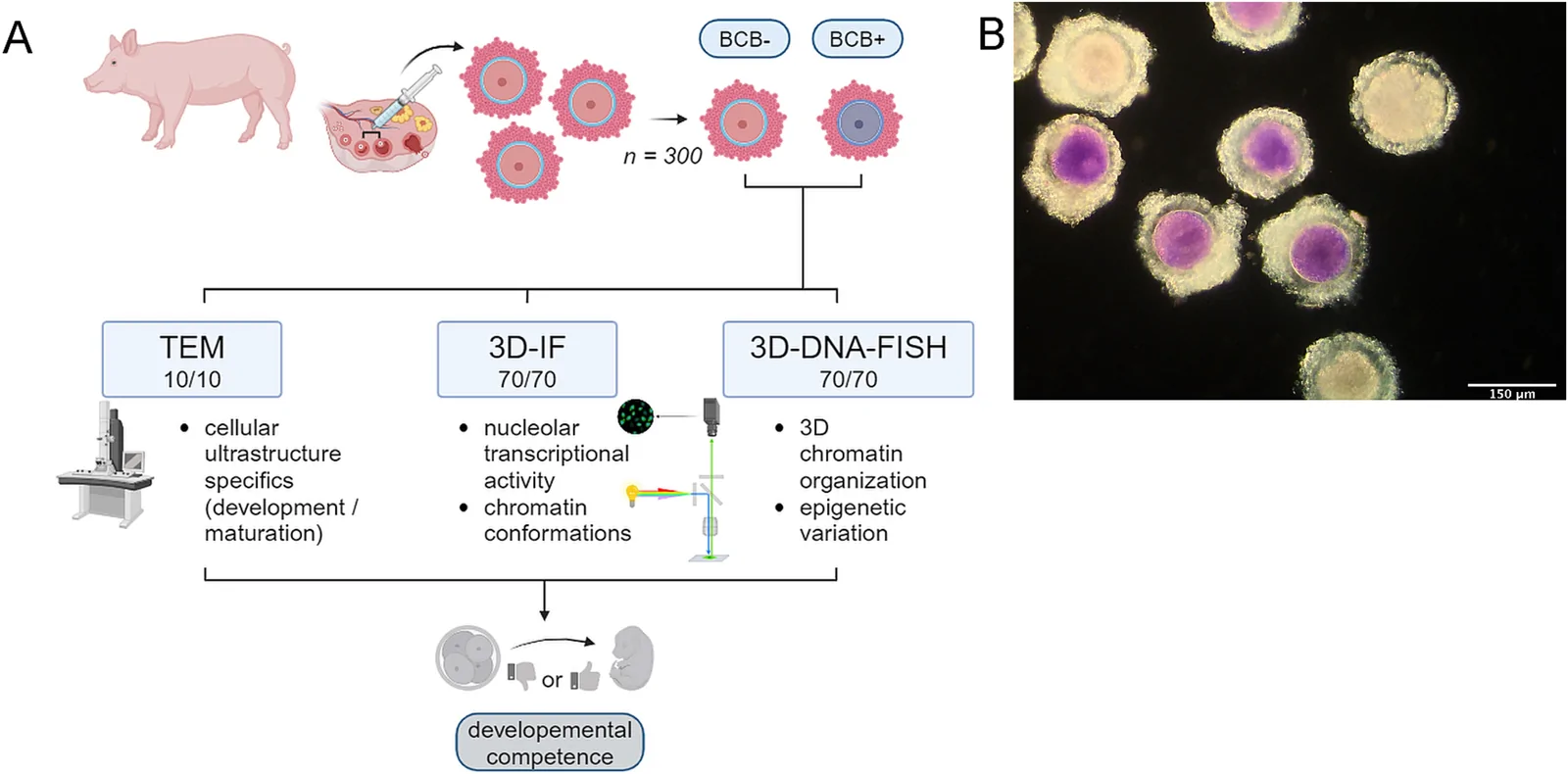
(A) Experimental design and analysis (Created in BioRender. Fenner, M. (2025) https://BioRender.com/t52w031); (B) Brilliant cresyl blue (BCB) staining as primary quality selector of this studies’ biological material (3D-preserved porcine cumulus-oocyte-complexes (COC)).

Finally, the oocytes were fixed for 15 min at RT in a fixative solution containing 100 μl PFA (15716, EMS), 25 μl triton X, 25 μl 0.5% PVP/PBS, 5 μl PMSF, and 345 μl PBS.

#### Transport

For transport the fixed oocytes were transferred to 2 ml Eppendorf tubes and stored in 0.5% PVP/PBS at 4°C until further processing.

#### Sample size

The initial sample size was 300 COC’s distributed into three groups for future reference (3D-IF, *n* = 140; 3D-DNA-FISH, *n* = 140; transmission electron microscopy (TEM), *n* = 20). Each group contained of two qualitative subgroups equal in numbers, as a result of the COC’s BCB-stain (BCB+ / BCB−).

### Immunofluorescence staining

#### Immunofluorescent labelling of UBF, CENP-A/-B, HP1b, H3K9me3, H4K20me3, and H3K4me3 in 3D-preserved porcine oocytes

Oocytes were permeabilized with 0.5% Triton X-100 in 0.5%PVP/PBS for 1 h at 37°C, saturated in 2% BSA for 1 h at RT, and further processed for 3D immunofluorescent staining.

The following primary antibodies were used at the indicated dilutions: Mouse monoclonal antibody against upstream binding factor (UBF) of the nucleolar transcription initiation complex (TIC) (Novus Cat# H00007343-M01, RRID:AB_548994; dilution 1:100), human CREST antibody which recognizes both centromeric protein (CENP)-A and -B (Erba Diagnostics Cat# HCT-0100, RRID:AB_2744669; dilution 1:250), mouse monoclonal antibody against heterochromatin protein 1 beta (HP1b, clone 1 MOD 1A9, Euromedex, dilution 1:250), and three rabbit polyclonal antibodies against histone modifications: H3K9me3 (Active Motif Cat# 39161, RRID:AB_2532132; dilution 1:500), H4K20me3 (Abcam ab9053, dilution 1:500), and H3K4me3 (Abcam 8550, dilution 1:200).

Oocytes from the respective BCB – and BCB + subgroups were incubated overnight at 4°C with the above mentioned primary antibodies, diluted in 2% BSA; washed three times for 10 min in 0.5% PVP/PBS at RT to remove any primary antibody excess, before final incubation with following secondary antibodies for 1 h at RT: Alexa Fluor 647-conjugated Donkey Anti-Mouse IgG antibody (Jackson ImmunoResearch Labs Cat# 715-605-151, RRID:AB_2340863; dilution 1:200), FITC-conjugated Donkey Anti-Rabbit IgG antibody (Jackson ImmunoResearch Labs Cat# 711-095-152, RRID:AB_2315776; dilution 1:200) or/and TRITC (rhodamine) – conjugated Donkey Anti-Human IgG antibody (Jackson ImmunoResearch Labs Cat# 709-025-149, RRID:AB_2340489; dilution 1:200).

Secondary antibody excess was removed by another three 10-min washing steps with 0.5% PVP/PBS before DNA counterstaining and mounting the oocytes with Vectashield (Vector Laboratories Cat# H-1000, RRID:AB_2336789) supplemented with 4′,6-diamidin-2-phenylindol (DAPI) (Thermo Fisher Scientific Cat# D3571, RRID:AB_2307445; dilution 1:100) on cover slips (SUPER FROST ULTRA PLUS, Menzel Gläser, Thermo Fisher Scientific).

#### Immunofluorescent labelling of UBF and H4K16ac combined with native EU staining in 3D-preserved porcine oocytes

Oocytes were cultured for 2.5 h in drops of modified FLI medium supplemented with 1 mM 5-ethynyl uridine (EU) (Invitrogen, cat.n. C10330) under mineral oil. The composition of modified FLI medium (REF) was: TCM199 medium supplemented with 10 IU/ml hCG (Prospec, HOR-250), 10 IU/ml PMSG (Prospec, HOR-272), 40 ng/ml FGF (Peprotech, 100-18B), 2 μl/ml LIF, 20 ng/ml IGF-1 (Peprotech, AF-100-11), 10 ng/ml EGF (Peprotech, A100-15), 6.85 mM L-glutamine, 0.57 mM L-cysteine, 50 μg/ml gentamicin, 0.2 mM sodium pyruvate, and 0.1% BSA. In the following the oocytes were fixed in 4% paraformaldehyde for 10 min at RT. Subsequently, the oocytes were permeabilized in 0.1% Triton X100 for 5 min at RT and rinsed with 1× PBS supplemented by 0.1% PVA. The oocytes were further processed by Click-iT RNA Imaging Kits (Invitrogen, cat. n.: C10330) in accordance with the manufacturer's instructions. Briefly, oocytes were further cultivated under the mineral oil in drops of Click-iT reaction cocktail for 1 h at RT. From that point on, the oocytes were kept in the dark. Subsequently, the oocytes were washed two times for 30 min in Component F (Click-iT RNA Imaging Kits). The following procedure was modified. Oocytes were blocked in 1× PBS supplemented with 3% BSA for 1 h at RT. Blocking was followed by culturing in primary antibodies diluted in 1× PBS supplemented with 1% BSA. A combination of EU with anti-UBTF (Novus Cat# H00007343-M01, dilution 1:400) and EU with anti-H4K16ac (Abcam cat. n. AB109463, dilution 1:200) were performed. Primary antibody incubation was performed overnight at 4°C. The following day, oocytes were rinsed three times for 20 min at RT in 1× PBS supplemented with 1% BSA and incubated in 1× PBS supplemented with 1% BSA and secondary antibody anti-mouse IgG (H + L) Alexa Fluor 488 (Sigma-Aldrich cat. n. 715-545-150, dilution 1:1000) for 1 hour at RT. Subsequently, the oocytes were rinsed three times for 20 min and mounted with Vectashield (Vector Laboratories Cat# H-1000, RRID:AB_2336789) supplemented with DAPI (Thermo Fisher Scientific Cat# D3571, RRID:AB_2307445; dilution 1:100).

#### Co-localization of BCB and DNA staining in in 3D-preserved porcine oocytes

BCB stained oocytes were rinsed in 1x PBS supplemented with 1% BSA and mounted with Vectashield (Vector Laboratories Cat# H-1000, RRID:AB_2336789) supplemented with DAPI (Thermo Fisher Scientific Cat# D3571, RRID:AB_2307445; dilution 1:100).

### 3D fluorescence in situ hybridization

3D DNA-FISH probes consisted of purified modified oligonucleotides labelled with either Cy5 or Cy3, specific for porcine meta- and acrocentric chromosomes: 5’-AGCGCTTGCCTAGTTCTCACCTAGC-3’ (Oligo SSCSR2A-Cy3, Eurogentec Cy → 3, MD-FL050–05004, metacentric) and 5-ATTCCATGCAGCGTGATTGA-‘3 (Oligo AC6-Cy5, Eurogentec Cy5→, MD-FL055-05004, acrocentric) [25]. The 3DFISH hybridization with metacentric and acrocentric probes was likewise performed on oocytes from the respective BCB – and BCB + subgroups. In brief, the zona pellucida (ZP) was removed by a rapid incubation in 0.1 N HCL for <10 s, followed by two brief washing steps in 0.5% PVP/PBS. The denuded oocytes were permeabilized 1 h in 10% triton X supplemented with 10% PVP, RNAse, and dissolved in PBS at RT, followed by another two 10-min washing steps. The permeabilized oocytes were then pre-hybridized with acrocentric and metacentric DNA FISH probes dissolved 1:10 in hybridization buffer (50% formamide, 2× SSC, 1× Denhart’s solution, 4 mM NaH_2_PO4 buffer and 10% dextran sulfate) for 3 h at 37°C. Denaturation was performed at 85°C for 10 min, whereafter the oocytes were placed in the hybridization oven at 37°C for at least 15 h. Finally un-hybridized probe remnants were removed by two 10-min washing steps with 2X SSC, 0.1% triton X-100, and 0.5% PVP diluted in H_2_O and the oocytes were parked in 0.5% PVP/PBS until further processing.

DNA counterstaining and mounting of the oocytes was performed as described in section Immunofluorescence staining.

### Transmission electron microscopy: Sample preparation

Porcine BCB + and BCB− oocytes were recovered and characterized as described in section Recovery of biological material. Primary fixation before transport, however, was performed with 3% glutaraldehyde (Merck, 1042390250) diluted in 0.1 M sodium phosphate buffer for 1 h at 4°C. The fixated samples were then stored and transported in 0.1 M sodium phosphate buffer at 4°C.

Immediately after transport, the oocytes were embedded in 4% Bacto agar (BD Diagnostics, 214010) at 45°C and forwarded to secondary fixation for 1 h in 1% OsO_4_ diluted in sodium phosphate buffer at RT. Before dehydration, the samples were well rinsed in MilliQ water. For dehydration the samples were subjected to a series of ascending ethanol concentrations (50% EtOH for 10 min, 70% EtOH for 10 min, 96% EtOH for 10 min, 99% EtOH for 3 x 20 min) followed by an intermediate solution consisting of propylene oxide (Merck, 8070271000) for 2 x 10 min. In the following, the samples were gradually infiltrated in Epon (812 Resin, TAABT031) until being left in pure Epon overnight in a rotator at RT. Embedding of the samples was finalized by polymerization of the Epon around the samples for 48 h at 60°C.

The cured epon blocks were subsequently cut in semi-thin 2 μm sections of the respective areas of interest, using an ultramicrotome (Reichert Ultracut S, Leica, Microsystems) fitted with a glass knife (LKB Bromma 7800, Leica Microsystems). Semi-thin sections were then stained with 1% toluidine blue (VWR 34187.185) diluted in 0.1% Borex (VWR 27727.231), where after sections of interest were selected by light microscopy for re-embedding [31]. Re-embedded sections were cut ultra-thin (50–60 nm), using the ultramicrotome fitted with a diamond knife (Jumdi, 2 mm). The ultra-thin sections were collected on 150 mesh copper grids (Gilder, G150-C3), coated with a parlodion-amyl acetate membrane (EMS), and contrasted with 2% uranyl acetate (Polyscience, 21447) and 1% lead citrate (Reynold, 1963).

### Observation with high resolution microscopy and image analysis

#### Confocal laser microscopy

Following immunofluorescent staining or 3D-DNA-FISH, respectively, the 3D-preserved oocytes were observed using a Carl Zeiss AxioObserver Zl fluorescence microscope equipped with a Zeiss LSM 510 confocal laser scanning microscope (MIMA2 Platform, INRAE and Faculty of Natural Sciences and Informatics, Constantine the Philosopher University in Nitra) and a corresponding Leica TCS SP5 II confocal laser scanning microscope (Leica Microsystems; Center for Advanced Bioimaging (CAB) Denmark, University of Copenhagen). The oocytes were visualized with an 63X oil (Zeiss) −/ water (Leica) -immersion objective. Z-stacks were acquired with a frame size of 512 × 512 or 1024 × 1024, a pixel depth of 8 bits, and a z-distance of 0.37 μm between optical sections. Wavelengths of 405, 488, 555, and 639 nm were used to excite DAPI, Alexa-488, or FITC, Cy3, and Cy5, respectively. Images consist of a stack of 40 to 80 z positions spaced of 0.37 μm and are in average of 22 μm of thickness.

#### Transmission electron microscopy

Transmission electron micrographs were acquired, using a Philips CM100 transmission electron microscope (Center for Advanced Bioimaging (CAB) Denmark, University of Copenhagen) equipped with an 11-megapixel Morada soft imaging system camera (Olympus) at 1950× and 10 500× magnification (Figure 2). Micrographs were qualitatively examined. Main ultrastructural features chosen for the oocytes’ maturity assessment were the presence or absence of the perivitelline space (PvS), quantity and morphology of microvilli and chromatin distribution within the nucleoplasm. Further assessed characteristics were lipid droplets, vesicles, and cortical granules.

**Figure 2:**
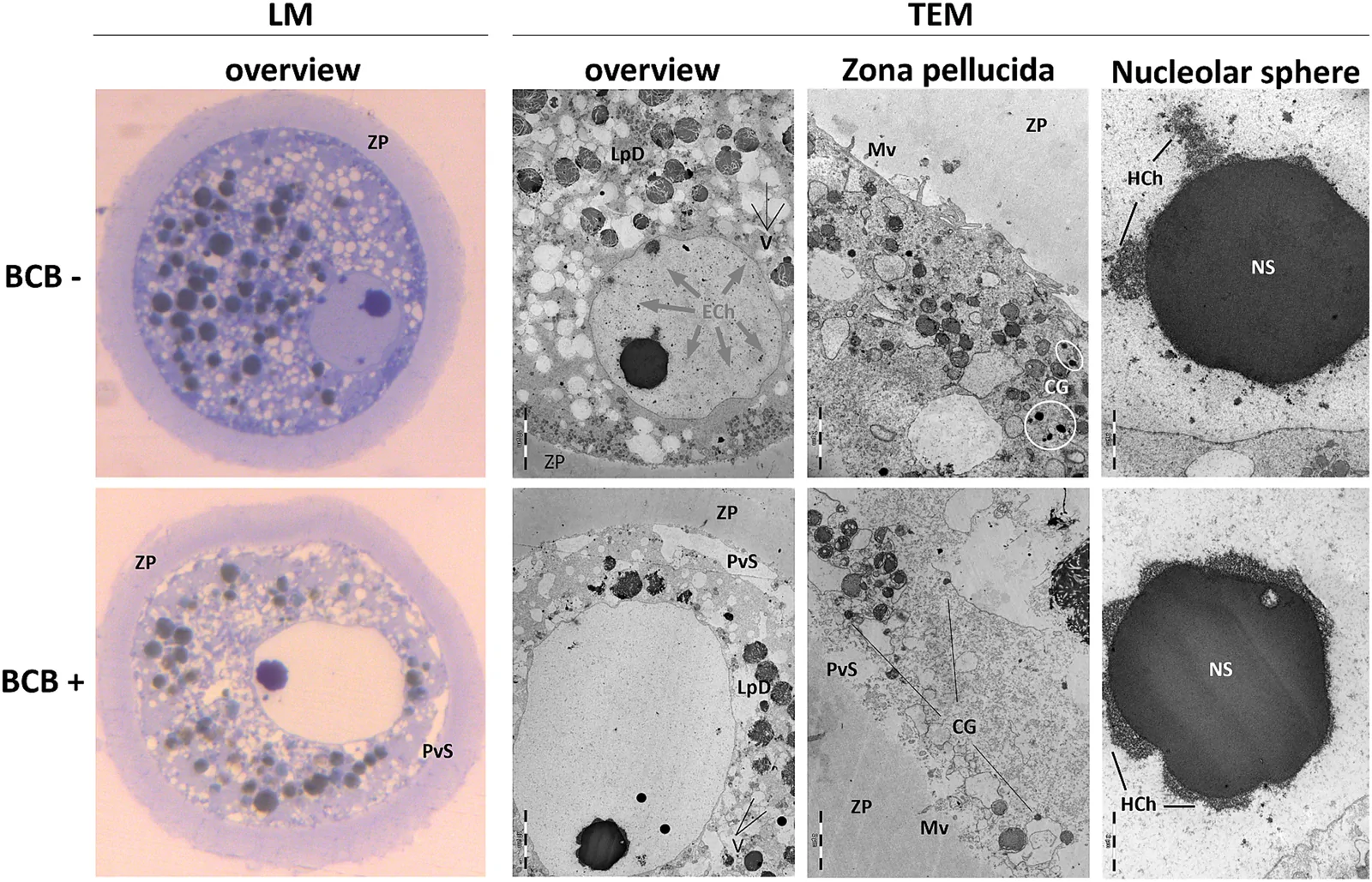
Qualitative ultrastructural validation of the biological materials’ developmental status: Light microscopic (LM) and transmission electron microscopy (TEM) images of BCB + vs. BCB− oocytes. LM overview: Semi-thin sections of selected BCB− and BCB+ oocytes stained with 1% toluidine blue; TEM: BCB−: narrow PvS, numerous erect microvilli (Mv) embedded into the zona pellucida (ZP), many clustered cortical granules (CG), presence of hetero- (HCh) as well as euchromatin (ECh, arrows) throughout the nucleoplasm; BCB+: moderate PvS, many microvilli erect into the PvS, solitary cortical granules and heterochromatin compaction around the nucleolar sphere (NS), absence of visible euchromatin in the remaining nucleoplasm (scale bar: overview 10 μm, ZP / NS 2 μm).

#### Overlay image production of co-localization of BCB and DNA staining

Light microscopy images were processed using a Carl Zeiss AxioObserver Zl fluorescence microscope equipped with phase contrast and ZEN Blue software. The DNA labelling was scanned using a Carl Zeiss AxioObserver Zl fluorescence microscope equipped with a Zeiss LSM 710 confocal laser scanning microscope with ZEN Black software.

#### Computational image analysis

Fluorescent profiles measurements were generated in Fiji [32–34] and 3D reconstructions using AMIRA 3.1 software (Mercury Computer Systems, Berlin, Germany, [35].

Quantitative image analysis was performed manually using Imaris 9.6 software (Oxford Instruments) available at the MIMA2 ISC INRAE (doi.org/10.15454/1.5572348210007727E12) as described by Chebrout et al [36]. Briefly, each channel was segmented using a threshold that allows discriminating specific fluorescent signals from the background. The nucleus segmented volume was used to select the segmented objects of channels corresponding to DNA-FISH signals of acrocentric and metacentric repeated sequences. The Imaris surface tool was used to measure the volume of the segmented objects.

### Statistical analysis

All data are presented as mean ± standard error of mean. Analyses were performed using GraphPad Prism 10 software (GraphPad Prism, RRID:SCR_002798) and R 3.3.1 software (R Project for Statistical Computing, RRID:SCR_001905), with *p* ≤ 0.05 considered significant. Statistical methods used, were unpaired t-tests and Fisher’s exact tests where appropriate and data was tested for normality using Shapiro–Wilk normality test.

## Results

This study employed porcine oocyte developmental biology as a translational model investigating epigenetics and 3D chromatin organization during oocyte growth and maturation. The study focused on an earlier proposed hypothesis that nucleolar transcriptional activity, nucleolar sphere compaction, and 3D chromatin organization are interrelated and contributes to the completion of oocyte growth.

A schematic representation of the experimental design and analysis is shown in Figure 1A.

### Oocyte cytoplasmic maturation displayed in cellular ultrastructure – Validation of the biological material

In order to validate the biological source material used in this study, oocytes were categorized into fully grown (BCB+) and still growing (BCB−) groups by BCB staining (Figure 1B). Subsequently, the cellular ultrastructure of these oocytes was assessed qualitatively by transmission electron microscopy (TEM).

The cellular ultrastructure of BCB+ oocytes, was distinguished by the presence of a moderate PvS, many microvilli erect into the PvS, solitary cortical granules and heterochromatin compaction around the nucleolar sphere (NS), as well as total heterochromatin absence in the remaining nucleoplasm (Figure 2). BCB− oocytes, however, were characterized by a very narrow PvS, numerous erect microvilli embedded into the zona pellucida (ZP), many clustered cortical granules and presence of hetero- as well as euchromatin throughout the nucleoplasm (Figure 2).

These distinct ultrastructural differences between BCB+ and BCB− oocytes supported BCB− staining as viable method for rough categorization regarding developmental status and thereby validated the utilized biological material as suitable for this study’ aims.

### Cytoplasmic maturation status associated to chromatin conformations – Timeline towards oocyte maturation

Oocytes from both the BCB+ and BCB− groups underwent integrated 3D-immunofluorescent staining including a DAPI-staining for DNA. Visualization of chromatin organization by DAPI in the growing oocytes at hand revealed the full range of the previously recognized chromatin conformations [24]: NSN, pNSN, pSN, and SN as well as their pre-condensed forms prematurely condensed pNSN (cpNSN) and prematurely condensed pSN (cpSN) (Figure 3). All these 3D chromatin conformation patterns were distributed between the BCB+ and BCB- group oocytes as depicted in Figure 3B at different frequencies. The BCB+ group contained a significantly higher number of oocytes displaying chromatin conformations categorized as fully grown (pSN, SN), whereas the opposite was true for the BCB− group (SN: 31/11, NSN: 5/20 (BCB+/BCB−); *p* < 0.0001). Both groups contained oocytes expressing prematurely condensed conformations (cNSN: 5/8, cpNSN: 11/12, cpSN: 11/13 (BCB+/BCB−); *p* > 0.05)) (Figure 3B).

**Figure 3:**
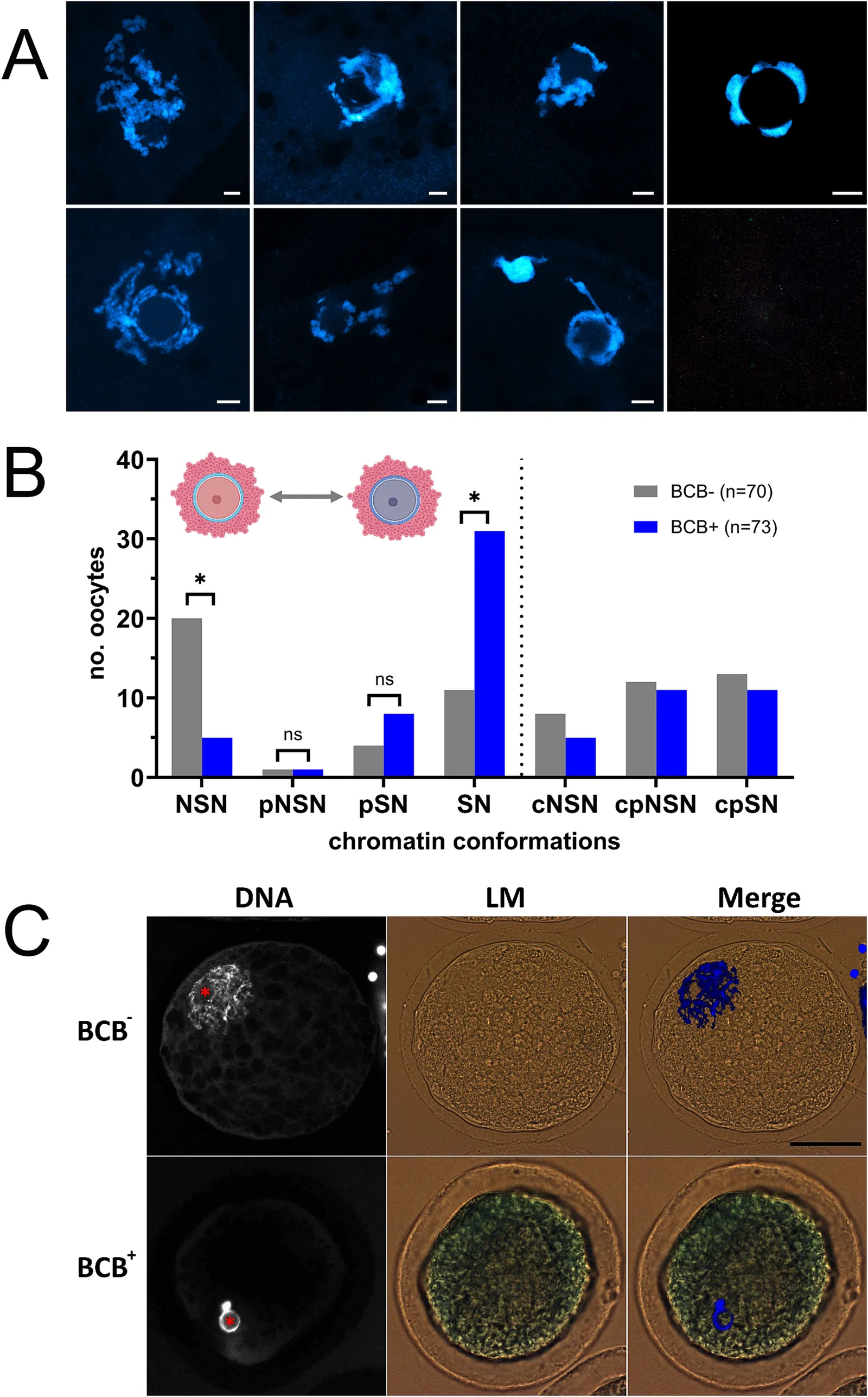
Cytoplasmic maturation status associated to chromatin conformations – timeline towards oocyte maturation; (A) Z-projections of 3D preserved oocytes corresponding to the chromatin configuration classification based on Pan et al. (DNA stained with DAPI = blue; white scale bar = 10 μm); (B) Total number of oocytes in each conformation category associated to cytoplasmic maturation status. (NSN: non-surrounded nucleolus, pNSN: partially non-surrounded nucleolus, pSN: partially surrounded nucleolus, SN: surrounded nucleolus, and prematurely condensed forms: cNSN, cpNSN and cpSN) (SN: 31/11, NSN: 5/20 (BCB+/BCB−); *p* < 0.0001); (C) Qualitative display of BCB- and DAPI-staining co-localization in BCB− (NSN) oocytes (upper panel) and BCB+ (SN) oocytes (lower panel) (red asterisks (^*^) marks the oocytes nucleolus; DNA stained with DAPI = bright blue in the nucleoplasm; BCB positive staining = blue–green cytoplasm; black scale bar = 50 μm).

The evident distribution of chromatin conformations in relation to the oocytes’ pre-categorization regarding cytoplasmic maturation status (Figure 3) supported the hypothesized interconnection of spatial changes in chromatin organization along a developmental timeline towards maturation.

In the following, results concerning prematurely condensed conformations (cNSN, cpNSN, and cpSN) will only be marginally reflected, as prematurely condensed forms only display in oocytes under non-favorable conditions, which render fertilization impossible [24].

### Linking oocyte cytoplasmic maturation and nucleolar transcriptional activity to chromatin conformation

As stated in the previous paragraph, BCB+ and BCB− oocytes were subjected to integrated 3D-immunofluorescent (IF) staining including upstream binding factor (UBF) as an integral part of the nucleolar transcription initiation complex (moreover, consisting of RNA polymerase I and promoter selectivity factor 1) [37], and detection of global RNA synthesis with native incorporation of 5-ethynyluridine (EU), staining newly transcribed RNA, to investigate the proposed link between nucleolar transcriptional activity and the growing oocytes developmental status (Figure 1 and Figure 4A and D). UBF-positive signalling, supported by positive EU labelling, was defined as a marker for nucleolar transcriptional activity.

**Figure 4:**
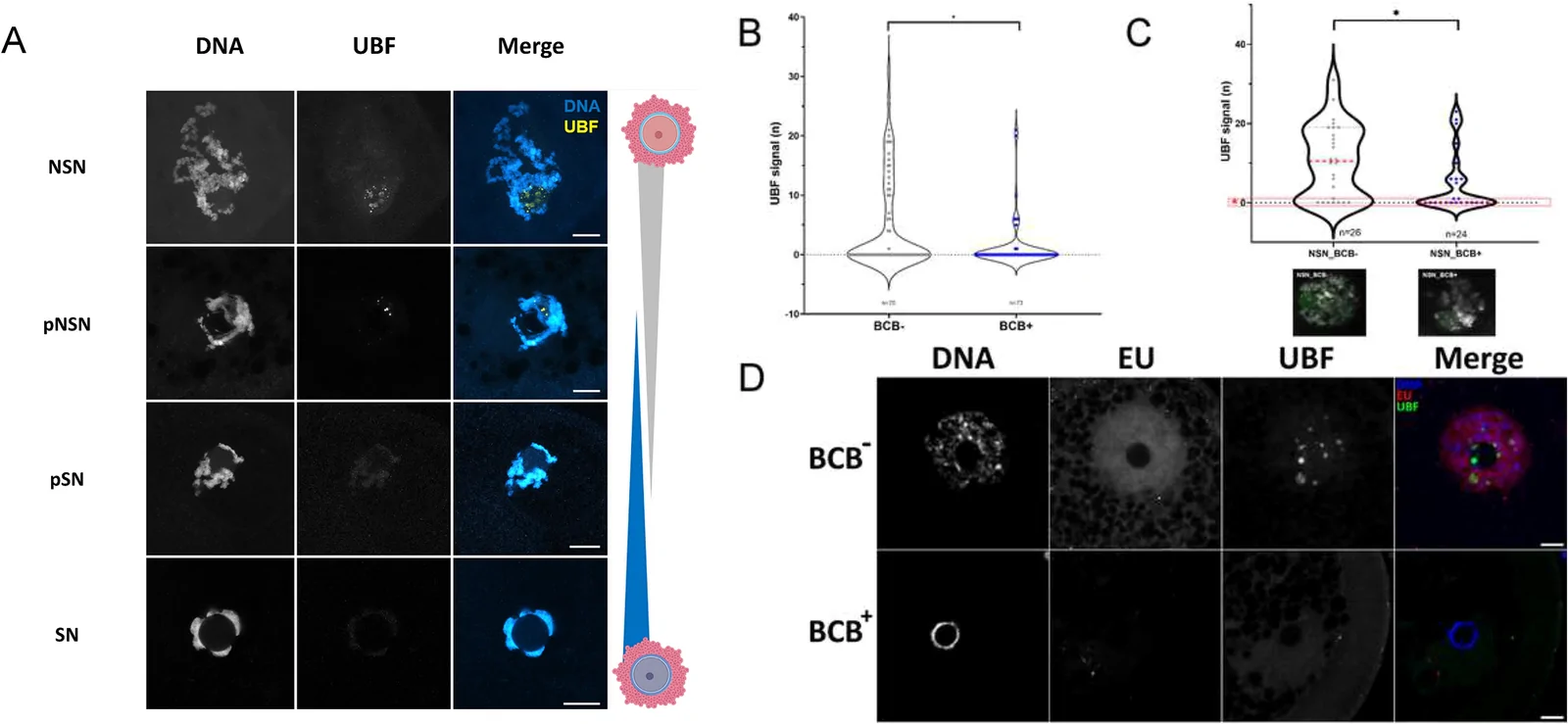
Linking oocyte cytoplasmic maturation and nucleolar transcriptional activity; (A) Z- projections of immunocytochemistry on 3D preserved oocytes (A) UBF pattern in the four main chromatin conformations as previously defined by Pan et al (UBF: yellow, DNA: blue, scale bar = 20 μm); (B) UBF-signal count from z-stacks of 3D-preserved oocytes: Significantly higher overall UBF signal count in BCB− (grey) than BCB+ (blue) oocytes. (C) UBF-signal count from z-stacks of 3D-preserved BCB± oocytes displaying NSN (summary of NSN, pNSN, cNSN, and cpNSN) chromatin conformation (−6 ± 2.4, *p* = 0.017). ^*^All NSN classified oocytes with zero UBF signal count were of premature condensed conformation (cNSN or cpNSN) (red dashed line: median); (D) Synchronized presence of EU and UBF labelling in BCB+ (SN) and BCB− (NSN) oocytes, displaying the interconnection between nucleolar transcriptional activity, oocyte cytoplasmic maturation and chromatin conformation. (EU: red, UBF: green, DNA: blue, scale bar = 10 μm).

UBF-signal foci (spots) were detected significantly more often in BCB− oocytes (*p* < 0.05; 4.8 ± 0.9 vs. 1.3 ± 0.5), albeit not exclusively (Figure 4A and B). However, a comparison between UBF-positive oocytes from the BCB− group and their respective counterparts from the BCB+ group revealed that UBF-positive oocytes in the BCB+ group exhibited a significantly lower number of signal foci (*p* < 0.05; 4.7 ± 1.5 vs. 10.7 ± 1.8) (Figure 4C). Hence, indicating gradual nucleolar transcriptional silencing towards final oocyte cytoplasmic maturation.

Conclusive analyses of the 3D-IF stainings revealed UBF-signal, and thereby transcriptional activity, only being present in NSN and pNSN oocytes (Figure 4A and C and Supplementary Figure S1B and C).

By defining an NSN-type including all oocytes potentially displaying UBF signal (NSN, pNSN, cNSN, and cpNSN), we were able to show, that nucleolar transcriptional activity is only present in growing oocytes under favorable conditions, as the prematurely condensed chromatin conformations showed minimal (cNSN) to none (cpNSN) UBF signal (Figure 4C and Supplementary Figure S1B). This clearly is in line with the above-mentioned observation, of a significantly lower UBF signal count in the BCB+ group, as most NSN-type oocytes (*n* = 22) in this group account for prematurely condensed cNSN (*n* = 5) and cpNSN (*n* = 11) conformations (Figure 3B).

Moreover, we discovered that the BCB+ group of SN oocytes (SN, pSN, and cpSN) only contained 25% (10 out of 40) prematurely condensed chromatin conformations, whereas the respective group categorized as BCB−, consisted of 50% (13 out of 26) prematurely condensed forms (Figure 3B).

Overall, less than a third (26%; 6 out of 23) of the NSN and none of the SN classified oocytes in the BCB+ group expressed UBF signal.

Additional dual immunofluorescent staining experiments were performed in both BCB+ (in SN configuration) and BCB− (in NSN configuration) oocytes with EU and post translational modification of histone 4 (H4K16ac) marking transcriptional activation. The gradual transcriptional silencing towards final oocyte cytoplasmic maturation (Figure 4) is concomitant with a spatial gathering of H4K16ac around the nucleolar sphere in the SN configuration (Figure 5). Of note, H3K4me3 (Supplementary Figure S2), a post-translational modification that marks active promoters, displayed a very similar spatial distribution behavior and is still present in transcriptionally silent SN-type oocytes.

**Figure 5:**
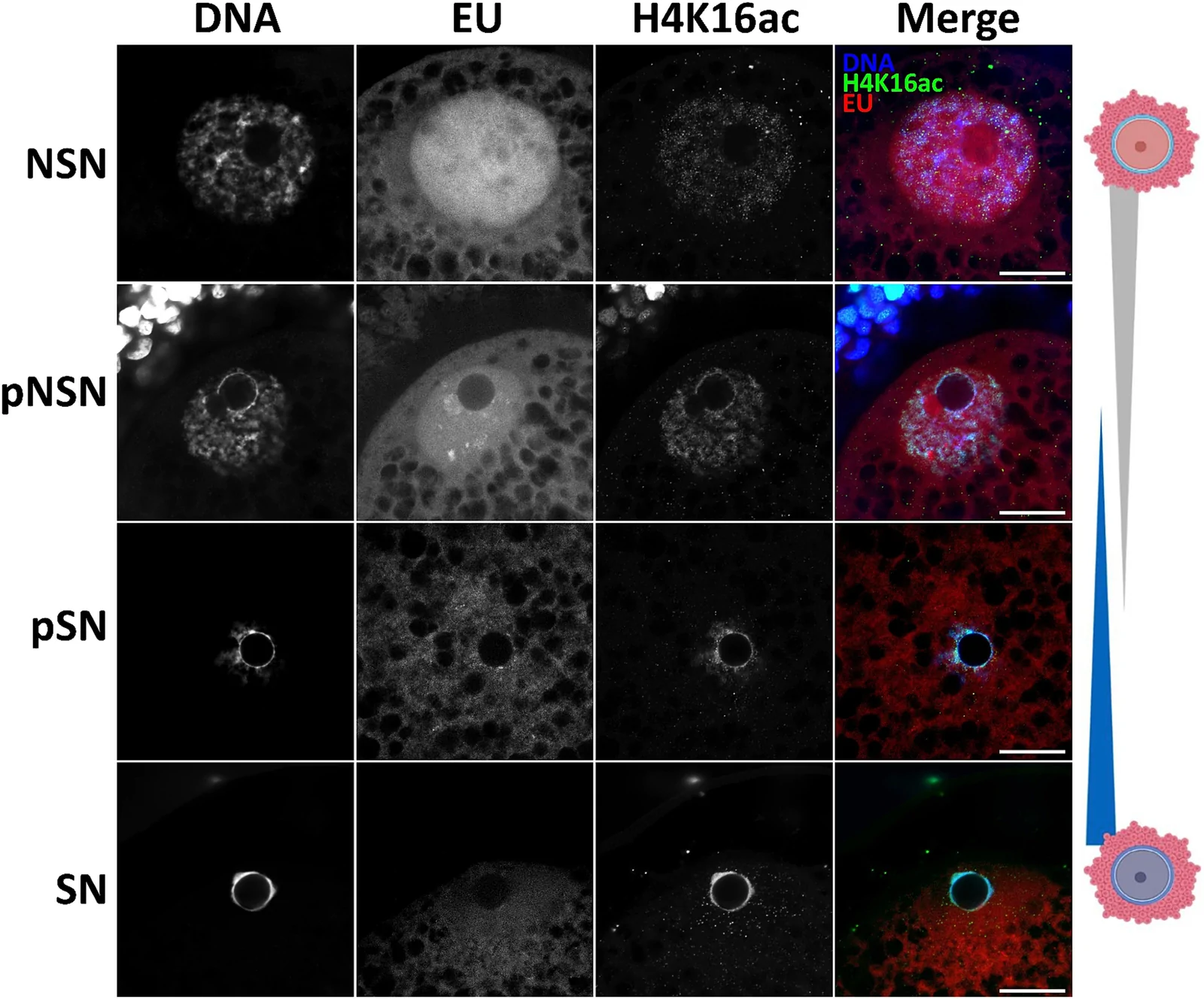
Synchronized relation between chromatin conformations, and nucleolar transcriptional activity (H4K16ac and EU) in growing porcine oocytes. Temporally and spatial co-localization of EU and H4K16ac signal following the changes in spatial organization of cytoplasmic DNA towards final oocyte growth. (EU: red, H4K16ac: green, DNA: blue, scale bar = 20 μm).

These findings hereby establish the initially hypothesized interconnection between nucleolar transcriptional activity, oocyte cytoplasmic maturation, and chromatin conformation, respectively.

### Changes in 3D chromatin organization

Having established a synchronized relation between chromatin conformations, and UBF expression, which is specific to nucleolar transcriptional activity in growing porcine oocytes, we aimed to further investigate the spatial heterochromatin organization of transcriptionally active or silent oocytes.

#### 3D-organization of centromeric and pericentromeric chromatin in growing porcine oocytes

To explore the spatial organization of heterochromatin linked to oocyte maturation status, we performed immuno-fluorescent detection of centromeric and pericentromeric chromatin regions, in oocytes displaying chromatin conformation patterns previously defined as fully grown (pSN, SN) and growing (NSN, pNSN). H3K9me3 and CENP A and B as well as HP1b and H4K20me3 served as markers for constitutive pericentromeric heterochromatin and active centromeres, respectively.

As expected, small centromeric spots (CENP) were observed to co-localize with pericentromeric DNA (H3K9me3) (Figure 6A and C). In NSN oocytes, pericentromeric regions appeared, in some cases, to co-localize with more than one centromeric spot, indicating close association of several chromosomes in so-called chromocenters (Figure 6C). Furthermore, these chromocenters were widespread throughout the nucleoplasm. Towards final maturation and transcriptional silencing of the oocytes (SN-type), the distribution of CENP- and H3K9me3-signal displayed distinct changes in its spatial organization, characterized by significant signal-clustering around the nucleolus (22.3 ± 1 vs. 14.8 ± 0.8; 21.7 ± 1 vs. 14.5 ± 0.8; Figure 6B), following the general chromatin conformation as displayed by DAPI-signal (DNA) (Figure 6C and Supplementary Figure S3). Display of identical patterns resulted from using HP1b for the labelling of pericentromeric regions and H4K20me3 for centromeres (Supplementary Figure S4). The HP1b signal labels the chromocenter like structure (as described for H3K9me3) with the presence of H4K20me3 signals observed as very small punctate dots (as described for CENPs signal) in NSN type oocytes. In SN-type oocytes, both HP1b and H4K20me3 signals are localized around the nucleolar sphere.

**Figure 6:**
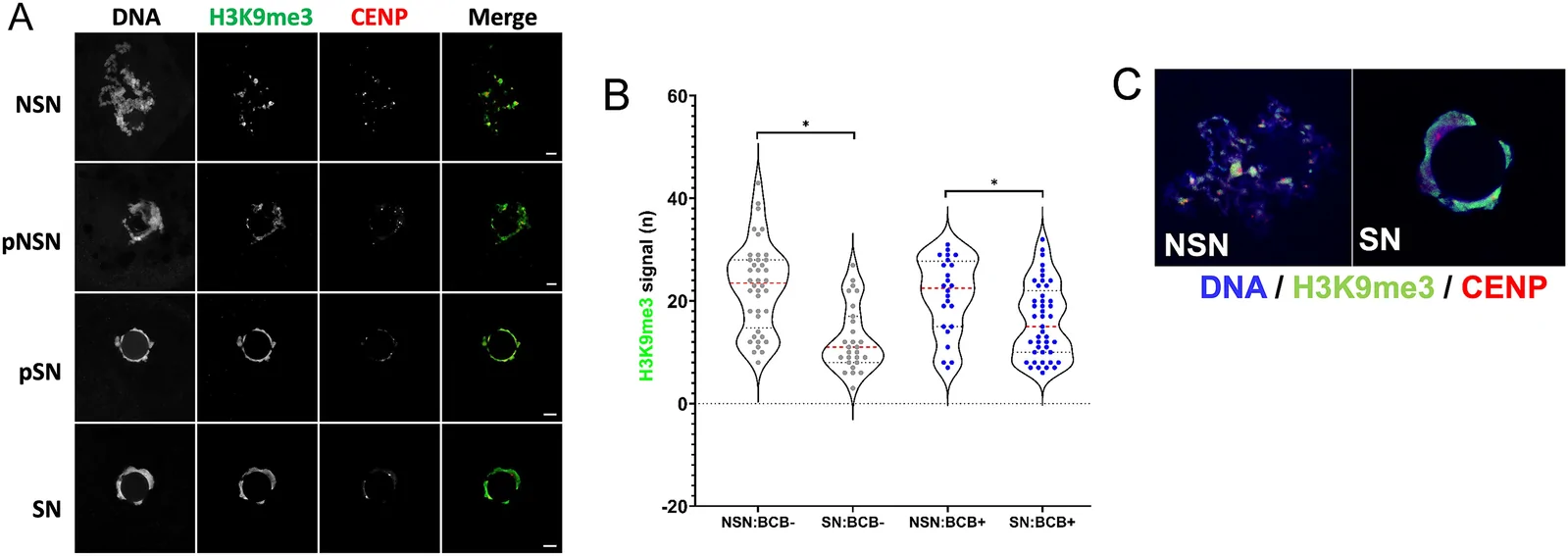
3D-organization of centromeric (CENP A/B) and pericentromeric (H3K9me3) chromatin in growing porcine oocytes; (A) Distribution of H3K9me3 (green) and CENP A/B (red) in the four chromatin configurations. (Scale bar = 5 μm.); (B) Violin-plots indicating the number of H3K9me3 signal counted in oocytes of respective chromosome configurations and cytoplasmic maturation stages (BCB+: −4933 ± 1853, *p* = 0.0097; BCB-: −10.21 ± 1954, *p* < 0.0001; red dashed line: median); (C) Images show a z-projection of NSN and SN oocytes.

#### 3D-organization of metacentric and acrocentric heterochromatin towards oocyte maturation

The centromere is defined as a region of specialized chromatin found within each constricted chromosome and in-depth investigation of the spatial organization of centromeric and pericentromeric DNA in growing oocytes was carried out by 3D-DNA-FISH.

The fluorescent signal representing metacentric repeated satellite sequences (Meta-SatRep) and acrocentric repeated satellite sequences (Acro-SatRep) [25], respectively, displayed overall the same behavior in terms of spatial distribution comparing NSN- and SN-type oocytes (Figure 7B and C) as shown for CENP, H4K20me3, H3K9me3, and HP1b (Figure 6C and Supplementary Figure S4). Localization of metacentric- and acrocentric pericentromeric heterochromatin changed from dispersed throughout the nucleoplasm in growing oocytes towards surrounding the nucleolus in their fully grown counterparts (Figure 7B and C). However, as the signal of Meta-SatReps outline was more delimited than that of H3K9me3 and HP1, 3D-DNA-FISH additionally revealed a significant decrease of the number of Meta-SatReps DNA-FISH spots (*p* = 0.0002; −5.7 ± 1.4) of pericentromeric heterochromatin around the nucleolar sphere in SN-classified oocytes of the BCB+ category compared to respective BCB− oocytes (Figure 7A).

**Figure 7:**
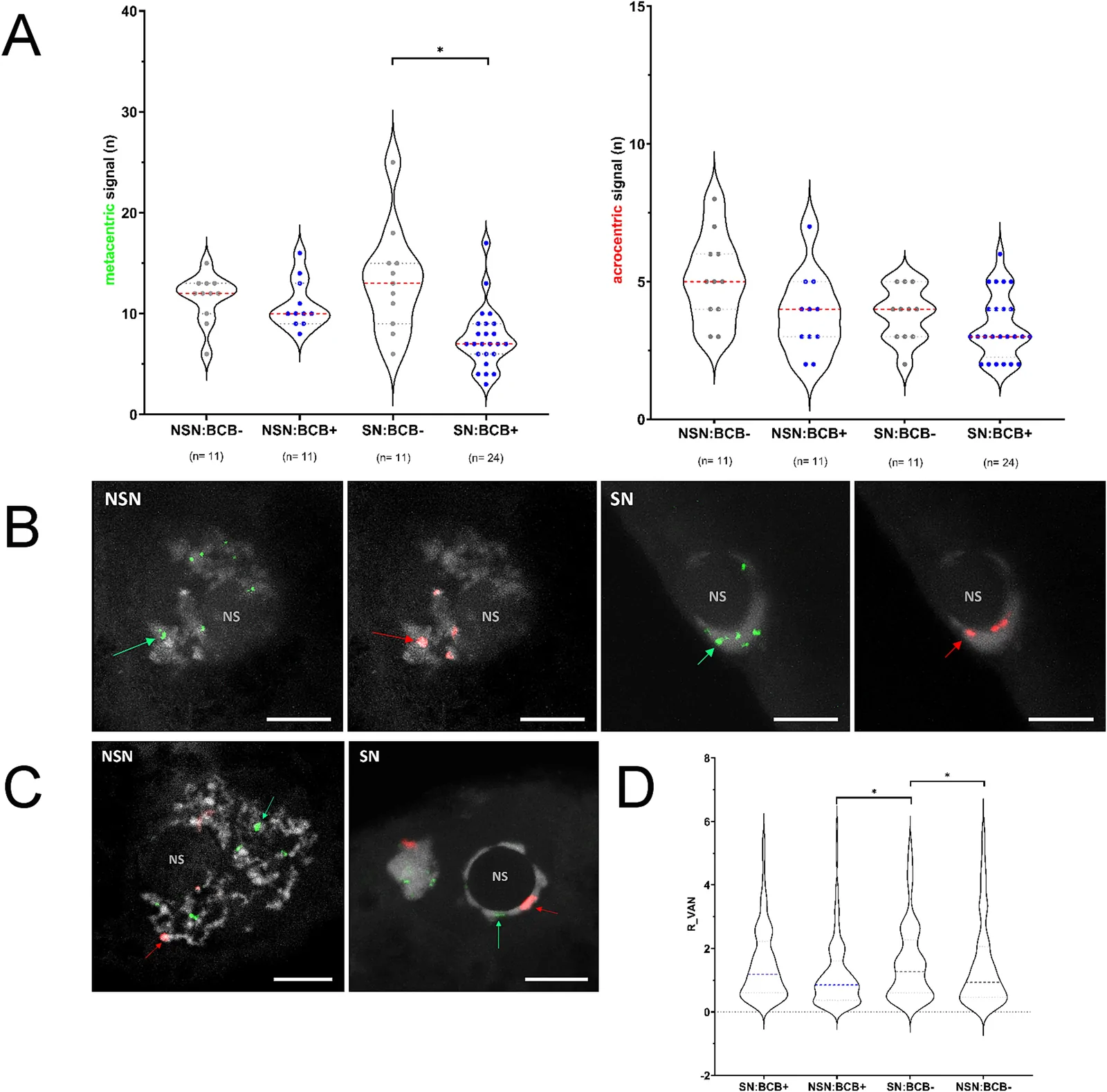
3D-organization of metacentric and acrocentric heterochromatin towards oocyte maturation; (A) Violin-plots indicate the number of SSCRS2A (meta) and AC6 (acro) signal counted in oocytes of respective chromosome configurations and cytoplasmic maturation stages (SN:BCB-/SN:BCB+: *p* = 0.0002); (B) Images show a z-projection of meta and acro signal in NSN (BCB−) and SN (BCB+) oocytes; (C) Merged distribution of META (green) and ACRO (red) signal in NSN and SN oocytes (Scale bar = 10 μm.); (D)Violin plots indicate the distribution of ratio between the volume of Meta-SatReps DNA-FISH spots and the volume of the DAPI-staining (nuclei) among the four categories of oocytes: NSN:BCB−/NSN:BCB+/SN:BCB−/SN:BCB+. (^*^ indicate *p* < 0.05).

This can be either associated with a higher state of compaction or a higher clustering of these pericentromeric repeated sequences in BCB+ SN oocytes. To test these two possibilities, we segmented 3D-DNA-FISH acrocentric, metacentric signals and DAPI staining using Imaris (as in Chebrout et al., 2023 [36]) and measured the volume of all objects identified. This analysis showed that the volume of the DAPI-staining, corresponding to the nucleus, is smaller in SN than in NSN (Supplementary Figure S5) and that the volume of Acro-SatReps DNA-FISH signal does not change whatever the chromatin configuration. On the other hand, Meta-SatReps DNA-FISH signal volume slightly increase in SN configuration. Because of the changes in volume of the nuclei between NSN and SN, we decided to consider the ratio between Meta-SatReps DNA-FISH signal and DAPI staining (Figure 7D). It shows that while the number of Meta-SatReps DNA-FISH spots decreases (Figure 7A), the volume increases, meaning that metacentric pericentric regions gather more and do not become more compacted in SN configuration oocytes.

Referring to the phenomenon of chromocenters, it can be noted that metacentric and acrocentric chromosomes did not co-localize yet formed type-pure clusters that were similar in structure to chromocenters (Figure 7C). In congruence with the porcine karyotype [38], (21 (sub)meta and 5 acro), the total number of countable (sub)metacentric signal was significantly higher than the acrocentric (Figure 7A).

## Discussion

Employing immunolocalization and high-resolution microscopy analyses of chromatin configurations, chromatin-specific epigenetic marks, and proteins specific for nucleolar activity in 3D-preserved oocytes, the present study addressed the apparent knowledge gap regarding epigenetic chromatin remodelling underlying porcine oocyte growth and maturation.

Commencing this studies’ investigations, the utilization of BSB staining to categorize porcine oocytes based on their cytoplasmic maturation status, was based on a comprehensive review of this methodology by Opiela et al [17]. Opiela et al. [17] emphasize, that the reported negative impact on RNA and protein content of the oocyte, which initiated the decade long controversial discussion of the BCB methodology, was mainly based on three studies, using either repeated [39, 40] or prolonged [41] incubation protocols with BCB dye. Using the standard BCB staining protocol (single incubation for 90 minutes at 38.5°C), however, Ergszegi et al. [14] reported superior meiotic progression, mitochondrial characteristics, and fertilization ability in BCB+ oocytes, compared to BCB− oocytes. Additionally, this methodology, employed for the purposes of this study, was validated by ultrastructural analysis, as previously described by P. Hyttel [42].

Recent studies, investigating porcine oocyte maturation and its link to oocyte quality and thereby developmental capacity in vitro, focused either solely on chromatin configuration as morphological marker for developmental potency [43], or transcription factor expression, related to later on embryonic morpho- and organogenesis regulation, in high- and low-quality oocytes, preselected by BCB-staining [15, 44]. We therefore decided to combine these two approaches and further investigate the evident knowledge gap between them. Is there a connection between cytoplasmic maturation (BCB ±), displayed chromatin conformations and epigenetic heterochromatin organization in porcine oocyte growth and maturation?

As previously stated, we employed a comprehensive classification for chromatin conformation in porcine GV oocytes [24] aligning nomenclature between model species [45], to facilitate direct comparisons between porcine and murine data regarding chromatin conformation and epigenetic maturation. Aiming for studying heterochromatin organization at high precision, we investigated the distribution of proteins (H3K9me3 and CENP) specific of heterochromatin—pericentromeric and centromeric regions, respectively [46], as well as of repeated satellite sequences (using oligonucleotides specific for porcine meta- and acrocentric chromosomes) playing a key role in remodeling and organization of the pericentromeric regions of the oocyte genome [23]. Furthermore, we utilized UBF and EU, as well as H4K16ac immunolocalization to be able to make a point regarding the oocytes’ transcriptional status [27, 28, 45, 47] regardless of its BCB classification.

In the process of oocyte growth, initially transcriptional activity increases, whereas it declines towards total silencing as the oocyte transitions from the growths phase to the fully-grown stage. This process is associated with a gradual change in spatial chromatin organization from NSN to SN configuration as demonstrated.

By coupling the oocytes’ BCB classification and displayed chromatin conformation with UBF signal presence, abundance and absence, as well as EU and H4K16ac signalling patterns, we were first and foremost able to validate the expected gradual nucleolar transcriptional silencing towards final oocyte maturation [24, 45] for the porcine species.

Amongst the BCB+ oocytes we not only found a significantly higher number of SN-type oocytes, as expected [43, 48, 49], but also a significantly lower UBF signal count. Confirmed by the complementary experiment combining EU and UBF co-localization, displaying a drastic decrease in signalling when changing from NSN to SN configuration, this conclusively supports this groups’ predominant maturity status.

Moreover, gradual accumulation of H4K16ac signal was observed, which peaked at the SN configuration as a remnant of the transcriptional activity during the oocytes’ growths phase.

Vice versa, we were able to show, that sufficing nucleolar transcriptional activity only is present in growing oocytes under favorable conditions, as prematurely condensed chromatin conformations showed minimal (cNSN) to none (cpNSN) UBF signal. This is consistent with the above reported observation, of a significantly lower UBF signal count in the BCB+ group, as most NSN-type oocytes (*n* = 24) in this group accounted for prematurely condensed cNSN (*n* = 4) and cpNSN (*n* = 15) conformations.

As previously established [24], premature condensation occurs under non-favorable conditions, leading us to hypothesize that the beforehand small proportion of SN oocytes observed in the BCB− group has experienced a premature condensation process as well and therefore expresses a state of condensation differing from their BCB+ equivalent (as shown in Figure 6B).

The observed chromatin conformations in relation to BCB pre-categorization supported the initial hypothesis that spatial chromatin changes align with the developmental timeline toward oocyte maturation [50]. The demonstrated ability of the BCB-stain to reliably distinguish between growing and fully grown oocytes with varying developmental competence further reinforces this link. Additionally, the correlation between chromatin organization and transcriptional activity highlights the pig’s value in translational research on oocyte quality and embryonic development, consistent with established findings in mice and humans [50].

Building on the confirmed relationship between chromatin conformation and nucleolar transcriptional activity (EU and UBF labelling) in growing porcine oocytes, we aimed to investigate the spatial chromatin organization underlying transcriptionally active or silent oocytes, with reference to previous published data in mice by Bonnet-Garnier et al [23].

In this previous study, post-translational histone modifications associated with chromatin permissive to transcription (i.e. H3K4me3 and H4K5ac) were described in NSN and SN-configuration oocytes. In pig, we found consistent results with this publication: a diffuse staining of both H3K4me3 and H4K16ac in NSN oocytes excluded from DAPI-dense regions. As in mice, these two permissive marks are still labelling the compact chromatin of SN oocytes although transcriptionally silent.

To the best of our knowledge, this study presents the first localization of pericentromeric (labelled by H3K9me3) and centromeric (labelled by CENP) regions in porcine oocytes. Furthermore, we identified chromocenter-formation [51], as previously found in mice and several other species [23, 51]. Immunolabelling of histone modifications (H3K9me3 and H4K20me3) specific for heterochromatin regions, revealed that their distribution in the nucleoplasm is very similar to previous findings in mice [23]. H3K9m3 and H4K20me3, exclusively labelling DAPI-dense regions, changed their organization from chromocenter-like to forming a ring around the nucleolar sphere as described in mice [23]. NSN-type oocytes display a less clustered organization of their satellite DNA repeats, than SN-type oocytes, as for pericentromeric regions of metacentric chromosomes formed bigger clusters in SN than in NSN oocytes. As in mice [23], the heterochromatin does not compact like the euchromatin during the change of chromatin conformation in growing oocytes but gathers and locates at the vicinity of the nucleolar sphere.

Further analysis of DNA satellite sequence organization demonstrated that porcine metacentric and acrocentric chromosomes do not co-localize yet form type-pure clusters that are similar in structure to chromocenters.

The specific function of chromocenters has yet to be uncovered, however, studies have documented their involvement in important processes such as genome encapsulation, gene expression regulation and speciation [51].

Studying the epigenetic maturation of porcine oocytes in respect of 3D-organization and spatial localization of chromatin and epigenetic marks linked to nucleolar activity, we conclude that we successfully established an evident interconnection between nucleolar transcriptional silencing during oocyte maturation and the display of specific spatial 3D-chromatin organization patterns.

Not only does this new information decrease the knowledge gap in porcine oocyte growth and maturation, underlining the translational value of the pig as model species in oocyte biology [50, 52], but validly propose changes in spatial localization of constitutive heterochromatin as morphological marker for developmental potency.

On the basis of the presented results, one could hypothesize that exclusively selecting BCB-positive SN-oocytes for IVM and subsequent IVF could potentially maximize successful outcome of embryonic development in vitro as it has been demonstrated in mice [19, 53, 54].

Transitioning in the optimal way on the timeline towards maturation, those oocytes seem to unite the unmitigated relation of cytoplasmic maturity, transcriptional silencing, and heterochromatin compaction, ensuring completion of maternal translational processes, resulting in sufficient accumulation of mRNA transcripts and proteins, prior to DNA compaction, which is prerequisite for successful initial embryonic development and embryonic genome activation [26].

The proposed sorting for BCB-positive SN-oocytes could potentially be realized by BCB- combined with Hoechst-staining. However, both claims call for further research – extending the material of interest from oocytes to embryos.

## Supplementary Material

Supplementary_figures_updated_ioaf098

## Data Availability

The data underlying this article will be shared on reasonable request to the corresponding author.
