# Supplementary material for: 3D-organization and spatial localization of chromatin and epigenetic marks linked to nucleolar activity in porcine oocytes
Source: Biol Reprod. 2025 May 3;113(4):ioaf098. doi: 10.1093/biolre/ioaf098 (PMC13738185; doi:10.1093/biolre/ioaf098)
Supplement: Supplementary file 1 [file supplementary_figures_updated_ioaf098.pdf]

Supplementary material:

Figure legends:

Figure S1: Oocyte cytoplasmic maturation and nucleolar transcriptional activity under unfavorable conditions; (a) Z- projections of immunocytochemistry on 3D preserved oocytes UBF pattern in three prematurely condensed chromatin conformations *(UBF: yellow, DNA: blue, scale bar = 20µm)*; **(b) UBF-signal count from z-stacks of 3D-preserved oocytes:** Significantly higher UBF signal count in NSN/pNSN conformations under favorable conditions, than in cNSN/cpNSN (unfavorable conditions) (BCB-: -14,74 ± 2,266, $p<0.0001$; BCB+: -9,214 ± 2,006, $p= 0.0002$); **(c) 3D modelling of UBF signal and chromatin conformations under favorable** (NSN/pNSN) **and unfavorable** (cpNSN) **conditions** *(created with IMARIS®)*

Figure S2: 3D-organization of histone 3 lysine 4 trimethylation (H3K4me3 post-translational modification marking active promoter regions; Z- projections of immunocytochemistry on 3D preserved oocytes H3K4me3 pattern following chromatin condensation from NSN to SN configuration *(H3K9me3: magenta, DNA: blue, scale bar = 20µm)*

Figure S3: 3D-organization of centromeric and pericentromeric chromatin in growing porcine oocytes under unfavourable conditions; (a) Images show z-projections oocytes in the three prematurely condensed chromatin configurations and the distribution of H3K9me3 (green) and CENP (red).*(Scale bar = 20µm)*

Figure S4: 3D-organization of centromeric and pericentromeric chromatin in growing porcine oocytes: Images show z-projections of oocytes in NSN and SN chromatin configurations and the distribution of H4K20me3 (green) and HP1b (red). *(Scale bar = 20µm)*

Figure S5: 3D-organization of metacentric and acrocentric heterochromatin towards oocyte maturation: Violin plots indicate the distribution among the four categories of oocytes: NSN:BCB-/NSN:BCB+/SN:BCB-/SN:BCB+ of **(A)** the volume of the DAPI-staining (nuclei); (B) the volume of Meta-SatReps DNA-FISH spots and (C) the volume of Acro-SatReps DNA-FISH spots.

Figure S1A:

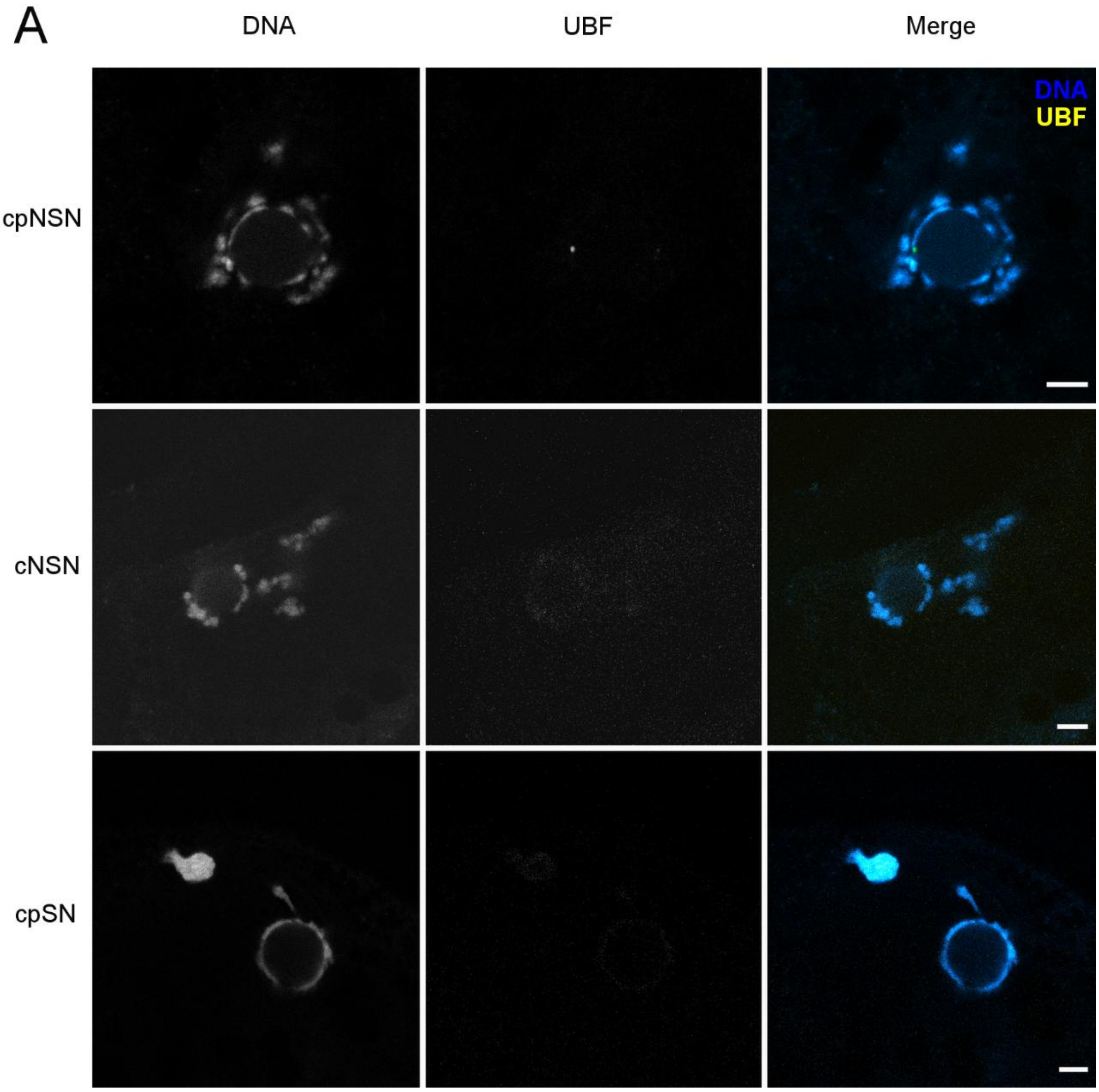

Figure S1 B and C:

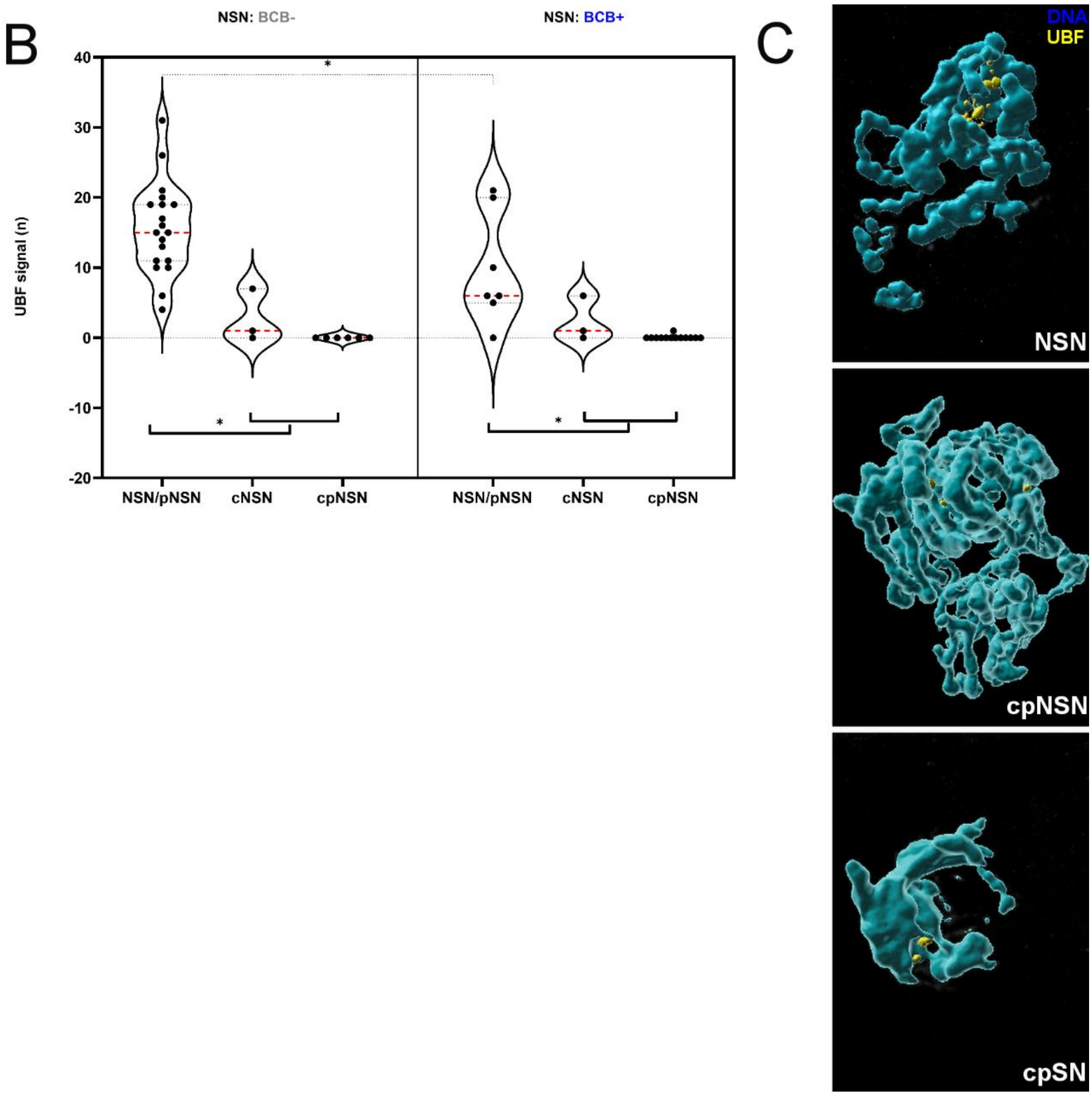

Figure S2:

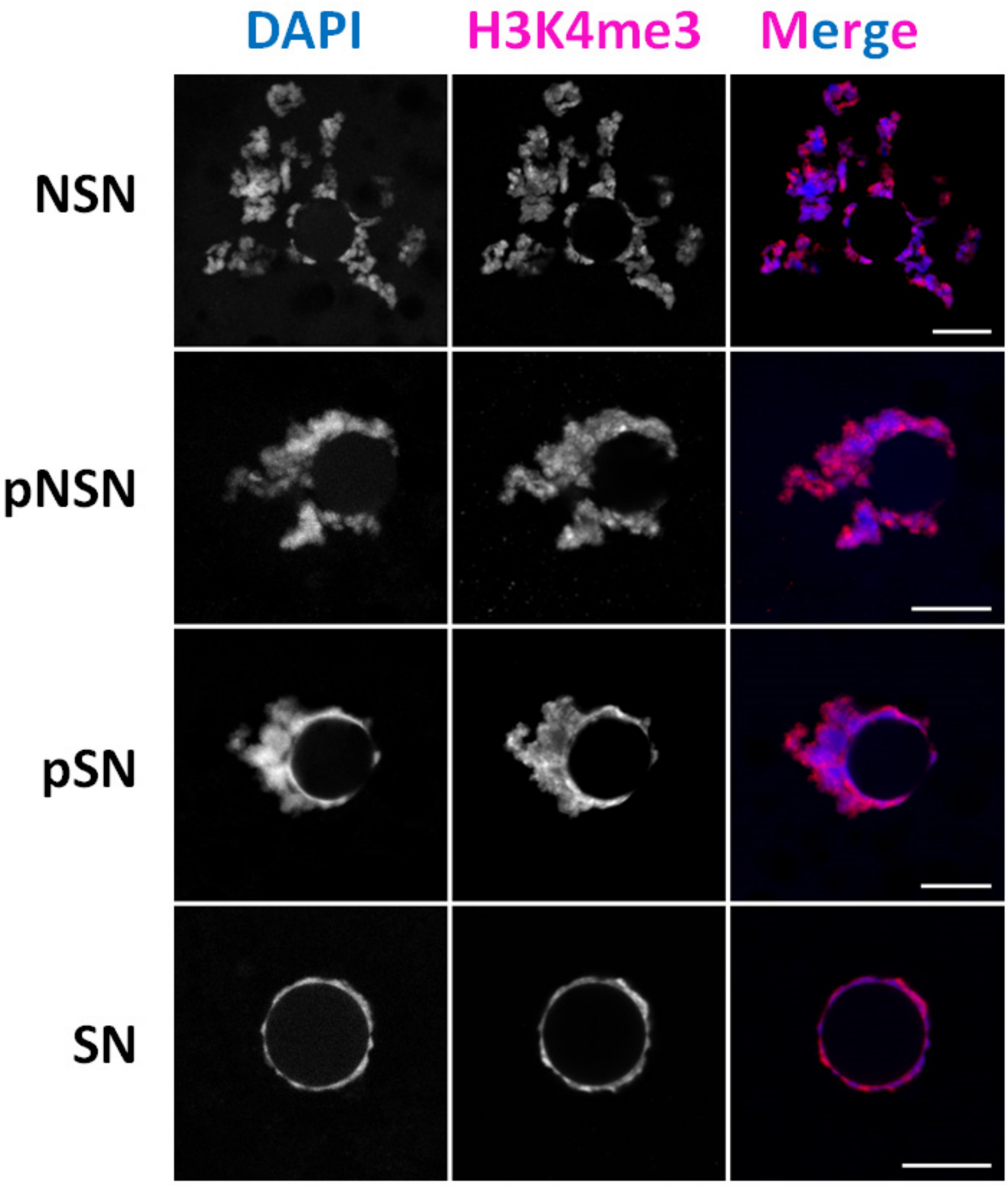

Figure S3:

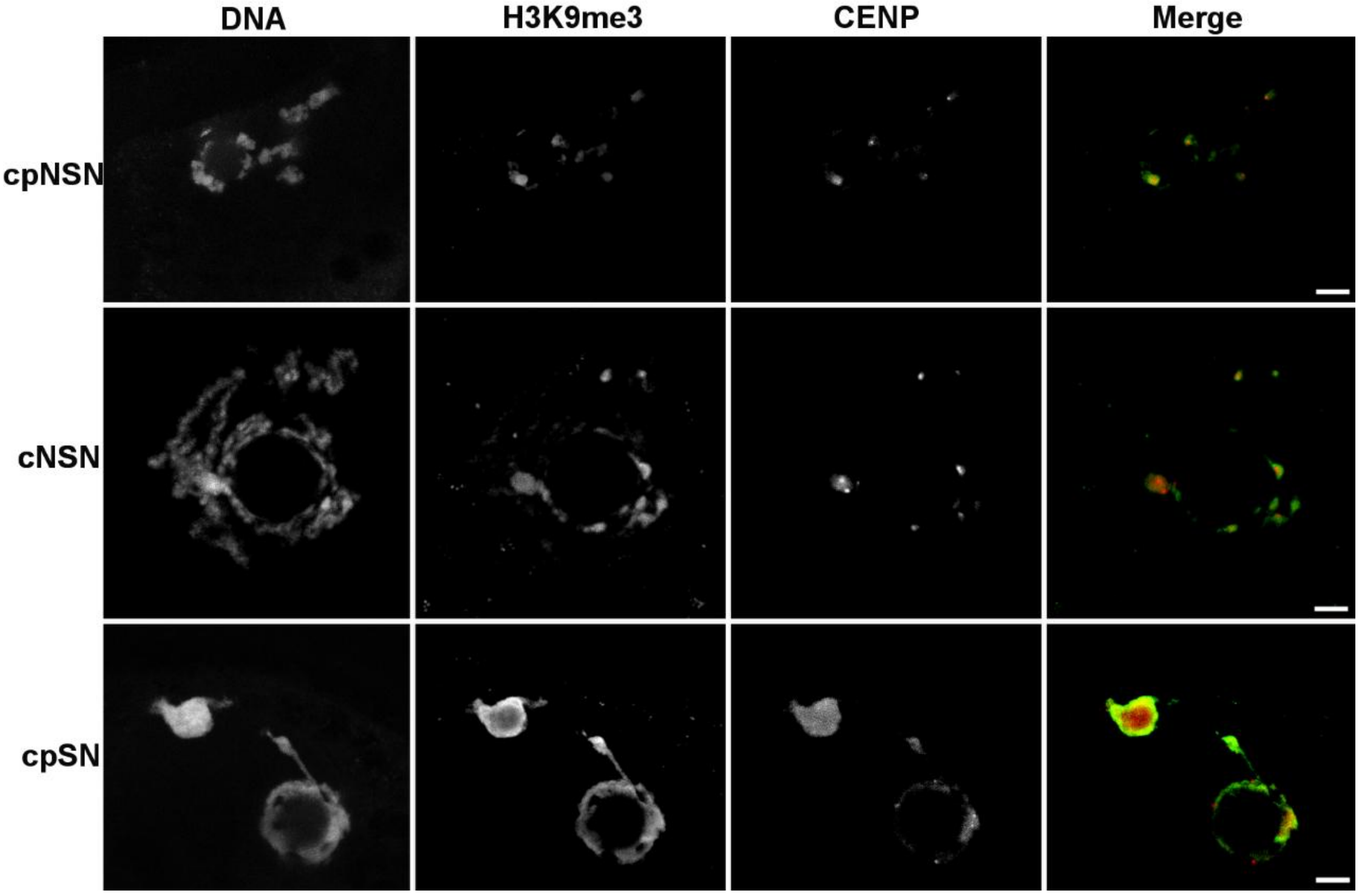

Figure S4:

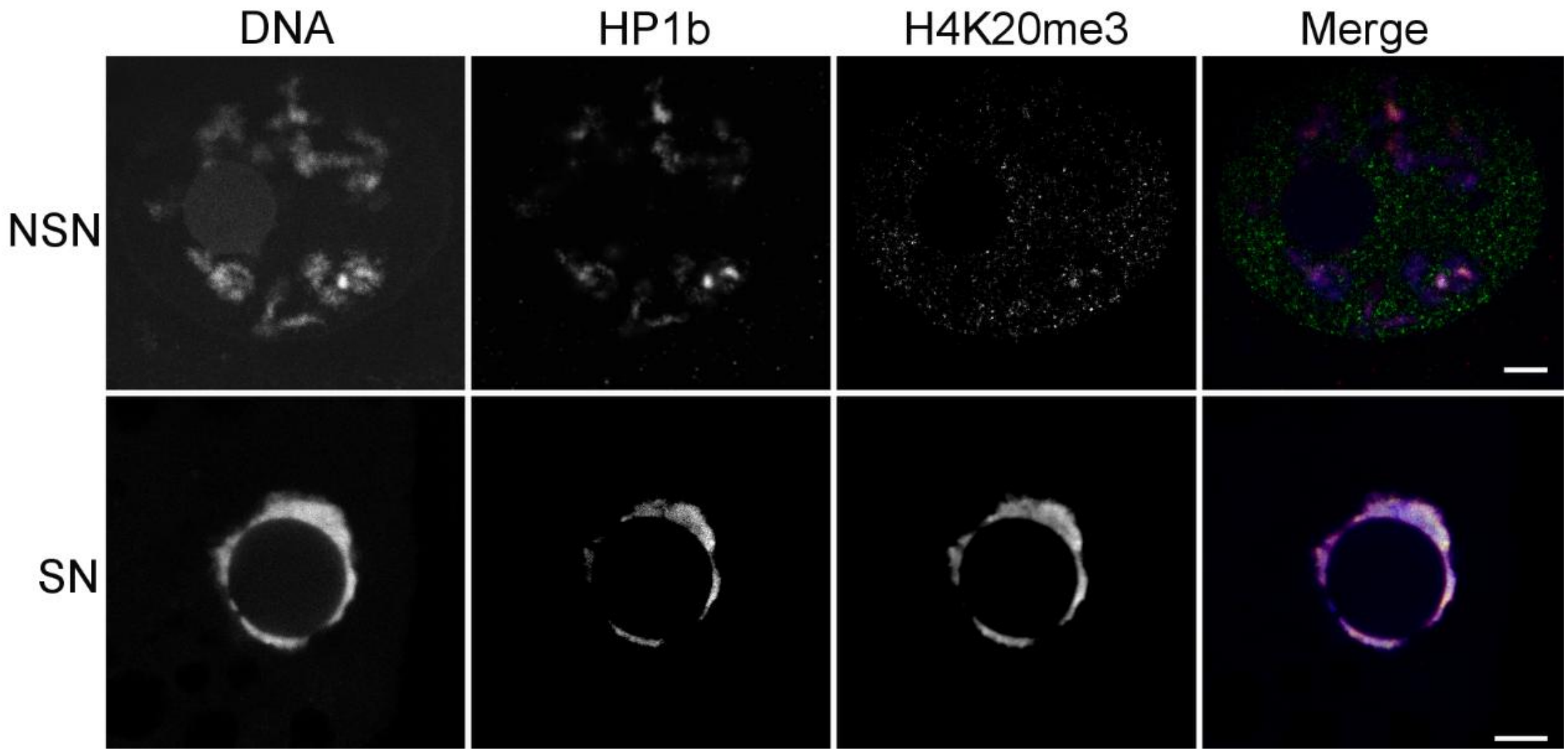

Figure S5:

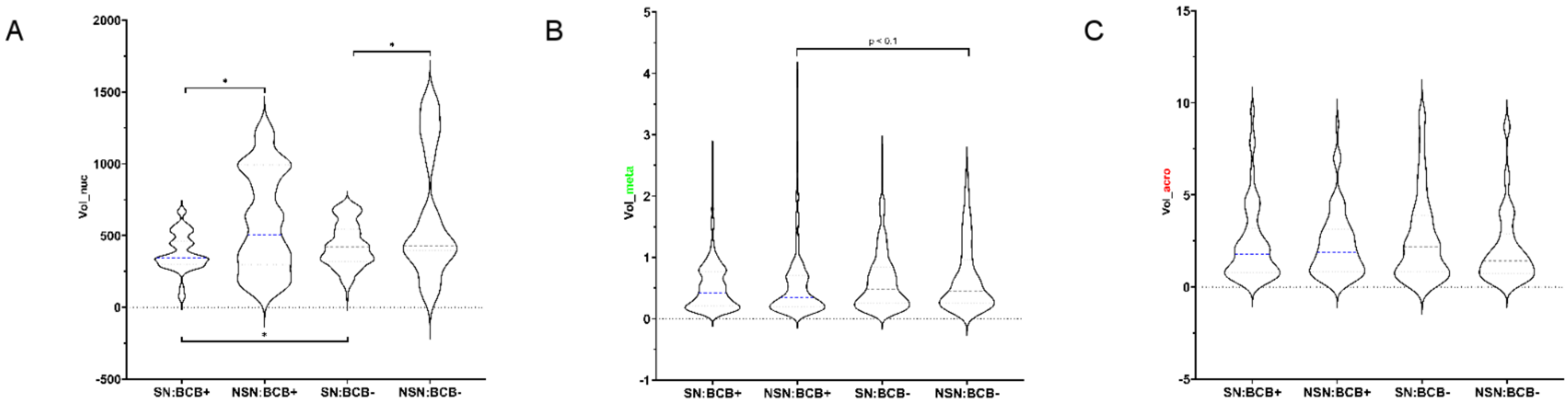
A
2000
1500
1000
500
0
-500
Vol_nuc
*
*
*
SN:BCB+
NSN:BCB+
SN:BCB-
NSN:BCB-
B
5
4
3
2
1
0
-1
Vol_meta
p < 0.1
SN:BCB+
NSN:BCB+
SN:BCB-
NSN:BCB-
C
15
10
5
0
-5
Vol_acro
SN:BCB+
NSN:BCB+
SN:BCB-
NSN:BCB-
